# To withhold or to implement bisphosphonate after cementless hip arthroplasty: a dilemma in elderly hip fracture patients

**DOI:** 10.1186/s13018-019-1104-2

**Published:** 2019-02-26

**Authors:** Young-Kyun Lee, Tae-Young Kim, Yong-Chan Ha, Kyung-Hoi Koo

**Affiliations:** 1 Konkuk University Medical Center, Department of Orthopaedic Surgery, School of Medicine, Konkuk University, 120-1, Neungdong-ro, Gwangjin-gu, Seoul, 05030 South Korea; 2 Department of Orthopaedic Surgery, College of Medicine, Chung-Ang University, 224-1 Heukseok-dong, Dongjak-gu, Seoul, 06973 South Korea; 3 Department of Orthopaedic Surgery, Seoul National University Bundang Hospital, 166 Gumi-ro, Bundang-gu, Seongnam, 13620 South Korea

**Keywords:** Bisphosphonate, Cementless hip arthroplasty, Subsidence, Stem migration, Femur neck fracture

## Abstract

**Background:**

Prior studies reported ambivalent effects of bisphosphonates on the fixation of cementless stem in hip arthroplasty patients. To set up the postoperative guide of bisphosphonate use after cementless hip arthroplasty, we investigated whether zoledronate has beneficial or negative effects in the stem migration and walking ability after cementless hemi-arthroplasty in elderly patients, who were operated due to femoral neck fracture.

**Methods:**

We compared 59 patients (zoledronate group), who received zoledronate after cementless hemi-arthroplasty, and 66 patients (control group), who did not receive that agent. We evaluated stem subsidence, cortical porosis around the stem, and walking ability with the use of Koval’s categories at 2-year follow-up.

**Results:**

No patient had more than 2 mm of stem subsidence in both groups. One patient in the control group had cortical porosis around the stem, but none in the zoledronate group. There were no significant differences in the postoperative Koval scores (*p* = 0.769) and in the proportion of walking recovery to pre-fracture status (*p* = 0.695) between the two groups.

**Conclusion:**

We did not find neither beneficial nor negative effect of this agent in terms of stem fixation and walking ability. Zoledronate can be used after cementless hemi-arthroplasty to manage the osteoporosis in elderly patients.

## Introduction

In elderly hip fracture patients, the treatment of osteoporosis, as well as that of the fracture, is important to prevent secondary fracture and other osteoporotic fracture [1, 2]. Bipolar hemi-arthroplasty (BHA) is a common treatment for hip fractures of elderly patients. Cementless BHA gained popularity because cemented prostheses are associated with a serious risk of fat embolization and hypotension during cementation [3, 4]. Prosthetic loosening is a cause of late failure after hip arthroplasty and remains a major concern, especially in elderly osteoporotic patients. Cementless femoral components are fixed by osseo-integration [5], and secure and solid initial stability is a key factor to promote the osseo-integration. The lack of initial fixation, early on, has been shown to result in stem migration, and finally in prosthetic loosening [6–10]. Old age and low bone mineral density were reported to adversely affect initial stability and to delay the osseo-integration of cementless stems [11].

Bisphosphonate is an anti-osteoclastic agent, which inhibits bone resorption. Several studies evaluated the effect of bisphosphonate on the fixation of cementless stem. However, these studies reported ambivalent effects. An early study, which was done in mongrel dogs, suggested cementless fixation of porous-coated implants were delayed or prevented by the administration of disodium etidronate [12]. A later study, which involved patients with femoral head osteonecrosis, showed that infusion of zoledronate improved initial fixation of a cementless stem by decreasing stem subsidence [13]. Another study reported risedronate, taken once weekly for 6 months following total hip arthroplasty, had no discernible effect on implant migration or clinical outcome in patients with osteoarthritis of the hip [14].

As far as we are aware, no study done to date has verified these three incompatible effects of the bisphosphonates on the stem stability. This issue should be sorted out, and the truth should be revealed to establish the postoperative protocols of bisphosphonate therapy after hip arthroplasty in elderly osteoporotic patients. If bisphosphonates have negative effects on the stem stability, the bisphosphonate treatment should be withheld or delayed until a bone-ingrown stability of the stem is achieved. However, if they have a beneficial or little effect, the treatment could be continued to manage the osteoporosis, irrespective of the surgical history.

The purpose of this study was to determine whether zoledronate has a beneficial, negative, or little effect in the stem migration and the outcome after cementless BHA in elderly patients, who were operated due to femoral neck fracture.

## Materials and methods

This study was approved by our Institutional Review Board (IRB number: B-1704/390-106), and informed consent was waived for this study.

### Patient selection

From March 2008 to February 2012, we retrospectively reviewed the 189 patients who underwent HA due to femoral neck fracture at our department. During this period, we implemented, exclusively, cementless stems and routinely recommended the use of zoledronate within 1 week after the BHA in osteoporotic patients.

The inclusion criteria for this study were as follows: patients who sustained the fracture due to a fall from standing or lower height, patients older than 65 years at the time of BHA, patients who had osteoporosis (BMD < − 2.5) at the diagnosis of hip fracture, patients who were ambulatory before fracture, and patients who were operated with single-design cementless stem (ML taper stem; Zimmer-Biomet, Warsaw, IN). Patients, who had a history of previous use of bisphosphonates and those who had femoral fracture or crack during the operation, were excluded. We also excluded patients who took other anti-osteoporotic agents than zoledronate after the BHA. Of the remaining 167 patients, 68 patients were infused with zoledronate after the BHA, while 99 patients refused to take zoledronate mainly due to high cost of the drug.

**Fig. 1:**
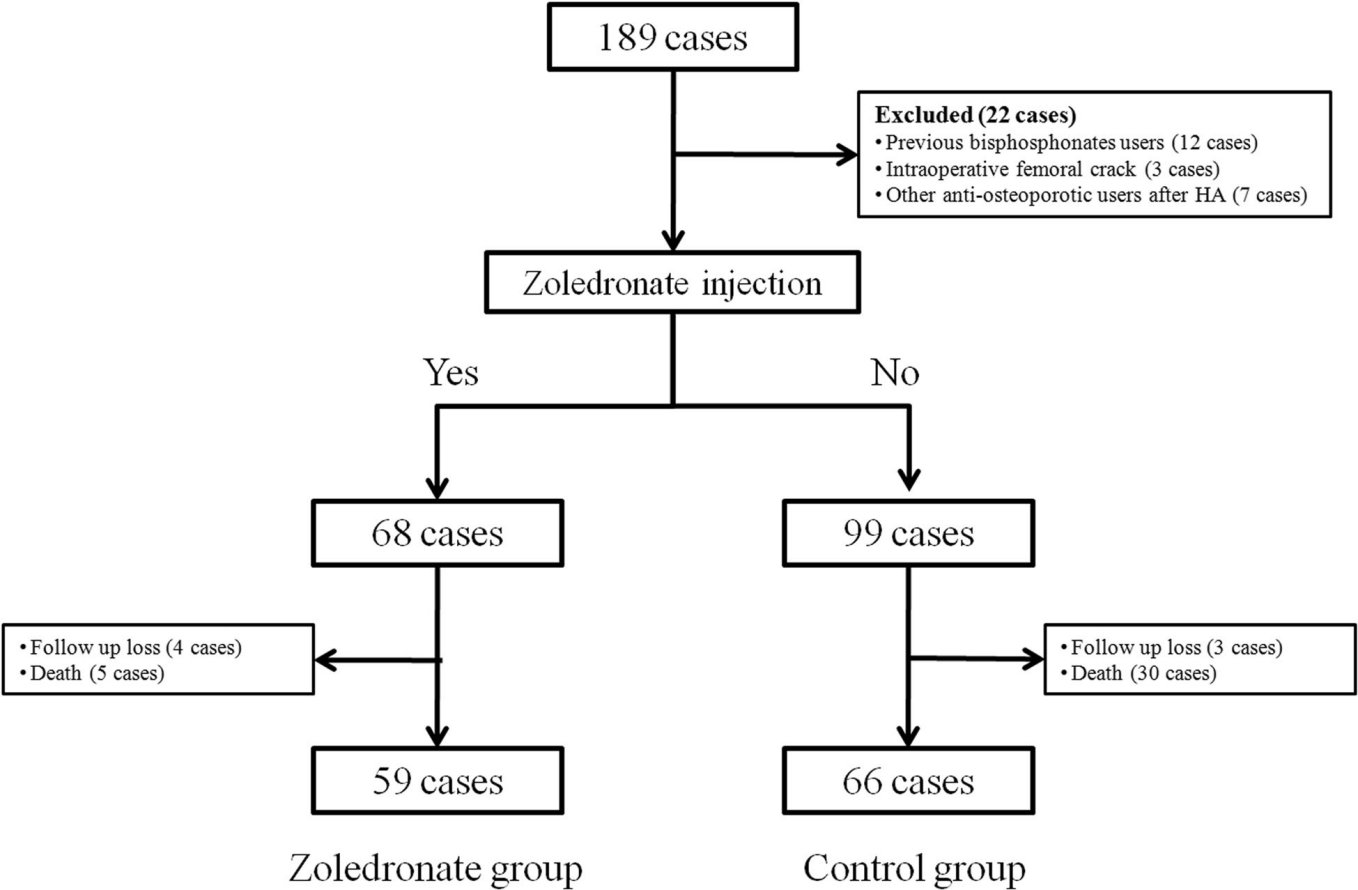
Flow diagram of patients’ selection in each group

Among the 167 patients, nine of zoledronate users and 33 of non-users were lost to follow-up or died before the 2-year follow-up after the surgery. Thus, 59 users (zoledronate group) and 66 non-users (control group) were included in the final analysis (Fig. 1).

None of the control group took zoledronate or any other anti-osteoporotic drug during the 2-year follow-up.

The zoledronate group included 43 women and 16 men with a mean age of 76.2 (65–93) years at the time of operation, and the control group included 40 women and 26 men with a mean age of 78.0 (65–94) years (Table 1). The average operative time was 75.0 (35–115) min in the zoledronate group and 73.0 (35–215) min in the control group. The average follow-up period was 4.5 (2.0–8.5) years in the zoledronate group and 4.0 (2.0–8.0) years in the control group.

**Table 1:** Characteristics between the zoledronate and control groups. Continuous variables were described as mean (SD)

	Zoledronate group (*n* = 59)	Control group (*n* = 66)	*p* value
Age (years)	76.2 (7.6)	78.0 (7.1)	0.182
Gender (cases)			0.147
Male	16	26	
Female	43	40	
Body mass index (kg/m^2^)	22.5 (3.3)	21.5 (3.1)	0.106
Bone mineral density (*t*-scores)	− 3.1 (1.2)	− 2.9 (1.3)	0.637
Facture sites (cases)			0.590
Right	35	36	
Left	24	30	
ASA (scores)	2.3 (0.6)	2.3 (0.7)	0.682
Anesthesia (cases)			
General	5	7	0.686
Spinal	54	59	
Operative time (min)	75.0 (17.1)	73.0 (25.3)	0.616
Femoral head (cases)			0.391
Metal	9	14	
Ceramic	50	52	
Follow-up (years)	4.5 (1.8)	4.0 (1.6)	0.122

There were no significant differences in age, gender, body mass index, bone mineral density, fracture’s site (right or left), type of anesthesia, operative time, used femoral head, or follow-up period between the two groups.

### Operative procedures

The femoral component was a tapered titanium stem. The stem had a plasma spray coating on the surface of the proximal one thirds. The bipolar cup is a cobalt-chromium cup (Self-Centering™ Bi-Polar; Zimmer-Biomet, Warsaw, IN), which contains a UHMWPE liner inside. A 28-mm metal head was used in 23 hips and the same size ceramic head in 102.

All operations were carried out through the postero-lateral approach by one surgeon. Rasps were used to shape the endosteal cavity of the proximal femur to the exact shape of the stem of the prosthesis. A stem trial was used to check the stem size, stability, and leg length. After the optimal stem size and head length were determined, the real stem was inserted into the femoral canal with a firm impaction.

One day after the operation, suction drainage was removed and patients were mobilized with tolerable weight-bearing and the use of assistive devices (wheelchair, walker, crutches, or cane). As their walking ability improved, their assistive devices were changed appropriately by a physical therapist.

Follow-up evaluations were performed at 6 weeks; at 3, 6, 9, and 12 months; and every year thereafter. Patients who did not visit the clinic were contacted by telephone questionnaire, and they were asked to send recent follow-up radiographs.

### Analysis of stem migration and postoperative outcome

The radiographic evaluation was done by two independent observers who did not participate in the operation. The 6-week antero-posterior and cross-table lateral radiographs were considered to be the baseline studies for radiographic comparison. The serial radiographic evaluation included an assessment of the stem subsidence and cortical porosis around the stem.

Stem subsidence was defined as “more than 2 mm change in the distance from the supero-lateral edge at the shoulder of the prosthesis to the tip of the greater trochanter” on the antero-posterior radiograph of the hip [15]. Cortical porosis was defined as a decrease in bone mineralization, resulting in a homogeneous, but somewhat sparse appearance of the remaining cortex around the stem [16].

The clinical evaluation was performed utilizing Koval’s categories for walking ability [17] at the final follow-up after surgery (Table 2). We also evaluated the recovery of walking ability to pre-fracture status.

**Table 2:** Categories of ambulatory ability

1	Independent community ambulators
2	Community ambulators with cane
3	Community ambulators with crutch or walker
4	Independent household ambulators
5	Household ambulators with cane
6	Household ambulators with crutch or walker
7	Nonfunctional ambulators

### Statistical analysis

For power analysis, 59 patients in each group would be needed with a power of 0.8 and type I error of 0.05 based on a prior study [13]. For statistical analysis, Student’s *t* test was used for continuous data, and chi-square test or Fisher’s exact test was used for categorical data. All reported *p* values were two-sided, and a *p* value < 0.05 was used to determine statistical significance. We used SPSS version 23.0 (Chicago, IL). The design and protocol of this retrospective study were approved by the institutional review board, who waived informed consent.

## Results

As of the latest follow-up, no patient in both groups had any stem subsidence (Table 3).

**Table 3:** Analysis of postoperative outcome and stem migration between zoledronate and control groups 2-year follow-up after surgery. Continuous variables were described as mean (SD)

	Zoledronate group (*n* = 59)	Control group (*n* = 66)	*p* value
Radiologic findings (cases)			
Stem subsidence	0	0	1.000
Cortical porosis around stem	0	1	1.000
Clinical findings			
Ambulatory status (Koval scores)			
Preoperative (A)	2.0 (1.6)	2.3 (1.7)	0.282
Postoperative (B)	3.2 (2.4)	3.7 (2.5)	0.325
Differences (B-A)	1.3 (2.2)	1.4 (2.5)	0.769
Recovery to pre-fracture status (cases)	43	46	0.695

One patient in the control group, and none in the zoledronate group, had cortical porosis around the stem.

Average differences of Koval scores before and after BHA were 1.3 in the zoledronate group and 1.4 in the control group, respectively (*p* = 0.769). There were no significant differences in the proportion of walking recovery to pre-fracture status (*p* = 0.695) between the two groups.

During the follow-up period, there was no joint infection or dislocation in either group.

## Discussions

Age-related bone loss of the proximal femur was reported to adversely affect the initial stability and delay osseo-integration after cementless hip arthroplasty [11, 18]. The effect of bisphosphonates on the fixation of cementless stem was ambivalent in prior studies [12–14, 19, 20].

Our study showed that stem migration did not occur after cementless BHA regardless of postoperative zoledronate treatment in elderly patients who were operated due to femoral neck fracture and their walking ability was not affected by this therapy.

Several studies reported the effects of bisphosphonates on the migration of cementless stem after hip arthroplasty. However, reported effects were quite different and equivocal in that regard. In 1987, Rivero et al. evaluated the effect of disodium etidronate (EHDP) on bone ingrowth of porous titanium composites [12]. EHDP was injected to six mongrel dogs for 8 weeks, and saline was injected to six additional dogs. After the first 4 weeks of treatment, the titanium composites were implanted into the humeri and the olecranons. After a total of 8 weeks of treatment, animals were sacrificed and the shear strength at the bone-porous implant interface was measured. The average shear strength in the EHDP group was reduced by 76% compared to that in the control group (*p* < 0.001). While ingrown bone was mineralized in all of the control specimens, the mineralization was inhibited in all of EHDP specimens. The authors suggest that bone ingrowth of cementless porous-coated implants may be delayed or prevented by EHDP. In 2009, Friedl et al. conducted a randomized clinical trial on 50 patients who underwent total hip arthroplasty due to femoral head osteonecrosis to evaluate the effect of zoledronic acid on early migration of cementless stem. They concluded that zoledronic acid improved the initial fixation of cementless implant as well as the clinical outcome [13]. However, 2 years later, Sköldenberg et al. performed a randomized clinical trial on 73 patients who underwent total hip arthroplasty due to osteoarthritis of the hip to investigate the effect of risedronate on periprosthetic bone resorption in the proximal femur after the arthroplasty. They reported that postoperative use of risedronate reduced the periprosthetic bone resorption up to 1 year after surgery, but had no discernible effect on clinical outcome and implant migration [14]. Friedl et al. counted any detectable subsidence as a stem migration and reported the stem migration of 0.91 mm in zoledronate and 1.2 mm in the control group at 2 years. In the study of Sköldenberg et al., the mean subsidence was 1.7 mm in both groups at 2 years. In both studies, the stem migration was measured with the use of Einzel-Bild-Roentgen-Analysis Femoral Component Analysis (EBRA-FCA) software (University of Innsbruck, Innsbruck, Austria) [9, 21]. It should be noted that the EBRA-FCA software has an accuracy of 1.5 mm, and it can detect a stem migration of > 1.0 mm with a specificity of 100% and a sensitivity of 78% [21]. Bieger et al. suggested that the migration of a femoral stem should not be assumed when a determined difference is less than 2 mm on plain radiographs [15]. In the current study, we counted the downward migration > 2 mm as stem subsidence.

In the current study, we could not find any differences in the postoperative Koval scores and in the proportion of walking recovery between the two groups. Friedl et al. reported that the Harris hip score was significantly more pronounced in patients treated with zoledronic acid at postoperative 2 years. However, thereafter, the score rapidly increased to an overall excellent outcome in both of the zoledronate and control groups.

While our study showed that the periprosthetic cortical porosis around the femoral stem was not different between the two groups, several studies showed that bisphosphonates reduced the periprosthetic bone loss. In a randomized trial, Scott et al. reported that zoledronic acid prevented loss of bone mineral density at 1 year and 2 years in Gruen zones 1 and 7 [22]. Sköldenbereg et al. reported that the mean bone mineral density in Gruen zones 1 and 7 was higher in the risedronate group than in the placebo group at 6 months and 1 year postoperatively [14]. However, they followed up these patients further and reported that the bone mineral density was similar in risedronate-treated group and control group at 4 years [23].

We note several limitations to this study. First, it was a retrospective comparison between zoledronate users and non-users. Zoledronic acid was solely approved for the treatment of osteoporosis. Thus, it was difficult to obtain approval for a prospective study to evaluate the effect of bisphosphonates on the stem fixation from the institutional review board. Nevertheless, stem subsidence did not occur in our patients who were treated with zoledronate. So, our study afforded sufficient evidence to substantiate that the use of the zoledronate has no negative effect on the stem migration. Second, a single design of cementless prosthesis was implanted to avoid implant-related confounding factors. Our results might not be otherwise if other prosthetic designs were used.

In conclusion, our study showed that stem subsidence did not occur in elderly patients after cementless BHA regardless of the use of the zoledronate and we did not find its negative effect in the walking ability. We therefore recommend the use of zoledronate after cementless BHA in elderly patients to manage the osteoporosis.

## Abbreviations

BHA: Bipolar hemi-arthroplasty
EBRA-FCA: Einzel-Bild-Roentgen-Analysis Femoral Component Analysis
EHDP: Disodium etidronate
